# Natural product databases: An annotated selection of World Wide Web sites relevant to the topics in *microbial biotechnology*

**DOI:** 10.1111/1751-7915.13295

**Published:** 2018-06-21

**Authors:** Lawrence P. Wackett

**Affiliations:** ^1^ Department of Biochemistry, Molecular Biology and Biophysics BioTechnology Institute University of Minnesota St. Paul MN 55108 USA

## antiSMASH bacterial version

http://antismash.secondarymetabolites.org

Bacterial antiSMASH is a computational tool for identification and analysis of secondary metabolic biosynthetic gene clusters in bacterial genomes.

## antiSMASH fungal version

https://fungismash.secondarymetabolites.org/#!/start

Fungal antiSMASH is a computational tool for identification and analysis of secondary metabolic biosynthetic gene clusters in fungal genomes.

## antiSMASH 4.0

https://www.ncbi.nlm.nih.gov/pubmed/28460038

This review article on antiSMASH describes improvements in chemistry prediction and gene cluster identifications.

## Computational approaches to mine for secondary metabolites

https://academic.oup.com/bib/advance-article/doi/10.1093/bib/bbx146/4590131

This article describes the use of antiSMASH databases for mining natural product gene clusters.

## RODEO for microbial biosynthetic potential

https://www.igb.illinois.edu/article/new-tool-rodeo-captures-breadth-microbial-biosynthetic-potential

RODEO is a tool for searching microbial genomes to identify natural product biosynthetic gene clusters.

## Beta‐lactone tool

http://z.umn.edu/bltool

This new database is focused on natural product beta‐lactone compounds. A growing number of beta‐lactone molecules are showing anticancer and antibiotic activities.

## Tools for biosynthetic gene cluster mining

http://www.secondarymetabolites.org/mining/

This page contains a compilation of secondary metabolite gene mining tools with links to the original databases.

## eSNaPD v.2.0

http://esnapd2.rockefeller.edu

eSNaPD stands for environmental Survey of Natural Product Diversity which is a tool to analyse crude metagenomic samples or bacterial culture collections for natural product gene clusters.

## NaPDoS

http://napdos.ucsd.edu

This is a tool for finding natural product genes, particularly those encoding C and KS domains.

## SANDPUMA

https://bitbucket.org/chevrm/sandpuma

This tool is focused on finding genes and proteins involved in the biosynthesis of non‐ribosomally synthesized peptides.

## ARTS

https://arts3.ziemertlab.com

ARTS stands for Antibiotic Resistant Target Seeker. It is a tool to screen secondary metabolites gene cluster for resistance genes.

## Fungal gene clustering

https://fungiminions.shinyapps.io/FunGeneClusterS/

This bioinformatics tool is focused on accurate prediction of secondary metabolite gene clusters in filamentous fungi.

## Promoter‐based prediction of gene clusters

https://academic.oup.com/bioinformatics/article/32/8/1138/1744063

This article describes a tools to search for islands of enriched cluster motifs and nearby anchor genes.

## PRISM 3

https://magarveylab.ca/prism/#!/prism

This tool accepts sequences and then searches genomes for encoded natural products.

## 2ndFind

http://biosyn.nih.go.jp/2ndfind/

This is a web‐based tool for finding natural product biosynthetic gene clusters.

## Beta‐lactamase database

http://www.bldb.eu

The beta‐lactamase database compiles information on sequences and structural biological information on beta‐lactamases.

## Antibiotic resistance database

https://card.mcmaster.ca

This antibiotic resistance database has information on genes, proteins and phenotypes.

## Super natural II database

http://bioinf-applied.charite.de/supernatural_new/

This database is very comprehensive, containing records of more than 300,000 natural products.

## NPASS

http://bidd2.nus.edu.sg/NPASS/

This database contains information on natural products, source organisms, biological targets and activities reports.

## ZINC

http://zinc.docking.org/browse/catalogs/natural-products

This site compiles links to information on natural product databases and commercial sources.

## PubChem

https://pubchem.ncbi.nlm.nih.gov

PubChem is a large database if chemical maintained by the National Center for Biotechnology Information. While it is not confined to natural products, there are many contained within, and it is strong on biological activity of molecules, which is particularly useful for natural products.

